# COVID‐19 Infection Confirmed by Bronchoalveolar Lavage Fluid Metagenomics ‐Next‐Generation Sequencing Instead of Pharyngeal Swabs in Follicular Lymphoma: Three‐Case Report and Literature Review

**DOI:** 10.1002/jcla.70103

**Published:** 2025-09-22

**Authors:** Can Liu, Yupeng Song, Siyan Niu, Yili Jiang, Tingting Zhu, Xin Li, Rui Cui, Qi Deng

**Affiliations:** ^1^ The First Central Clinical College, Tianjin Medical University Tianjin China; ^2^ Changchun University of Chinese Medicine Changchun China; ^3^ Department of Hematology Tianjin First Central Hospital, School of Medicine, Nankai University Tianjin China

**Keywords:** bronchoalveolar lavage fluid, COVID‐19 infection, follicular lymphoma, metagenomics next‐generation sequencing

## Abstract

**Background:**

Hematologic malignancy patients with B lymphocytopenia after anti‐CD20 monoclonal antibody or anti‐CD19 chimeric antigen receptor (CAR) T cell therapy often face prolonged SARS‐CoV‐2 positivity on pharyngeal swabs and persistent or recurrent COVID‐19 infection, resulting in high mortality.

**Methods:**

Here, we describe three follicular lymphoma (FL) patients with persistent fever, cough, and hypoxemia, but they were ruled out for bacterial, viral, fungal, and other pathogen infections, and the throat swabs were consistently SARS‐CoV‐2 negative. These FL patients with B lymphocyte deficiency who were diagnosed with COVID‐19 infection confirmed by bronchoalveolar lavage fluid (BALF) metagenomics next‐generation sequencing (mNGS). Their COVID‐19 infection was characterized by differences in viral load in the upper and lower respiratory tracts. When this particular COVID‐19 infection occurred, although their percentages and absolute values of CD8^+^ T cells and CD4^+^ T cells were normal, they all had B lymphocyte deficiency and hypogammaglobulinemia. They all had low expression of interleukin (IL)‐6 in peripheral blood inconsistent with clinical infection symptoms.

**Results:**

The patients received a combination therapy of molnupiravir and methylprednisolone; then their symptoms were relieved over the next 2 weeks–2 months.

**Conclusion:**

Therefore, for immunocompromised patients, especially those with B lymphocyte deficiency, hypogammaglobulinemia, and low expression of IL‐6 in peripheral blood inconsistent with clinical infection symptoms, mNGS for BALF should be performed as soon as possible in this particular condition to confirm the diagnosis of COVID‐19 infection.

## 1 Introduction

The novel coronavirus, severe acute respiratory syndrome coronavirus 2 (SARS‐CoV‐2), has led to high rates of morbidity. There was a mortality rate ranging from 31% to 35% in non‐Hodgkin's lymphoma during the COVID‐19 infection pandemic. Furthermore, persistent viral infection > 6 weeks was associated with high mortality [1, 2]. Patients with hematologic malignancies with B lymphocytopenia are an important group of patients with persistent COVID‐19 infection [3]. Prolonged SARS‐CoV‐2 positive on pharyngeal swabs and persistent or recurrent COVID‐19 infection have been reported in lymphoma patients with B lymphocyte deficiency, especially those treated with anti‐CD20 monoclonal antibody or CD19 CAR‐T cell therapy [4, 5]. Here we show three patients with follicular lymphoma (FL) after such therapy who were diagnosed with COVID‐19 infection confirmed by metagenomics next‐generation sequencing (mNGS) of bronchoalveolar lavage fluid (BALF), but they all had persistently negative results for SARS‐CoV‐2 on pharyngeal swabs.

## 2 Case Presentation

### 2.1 Case 1

**FIGURE 1:**
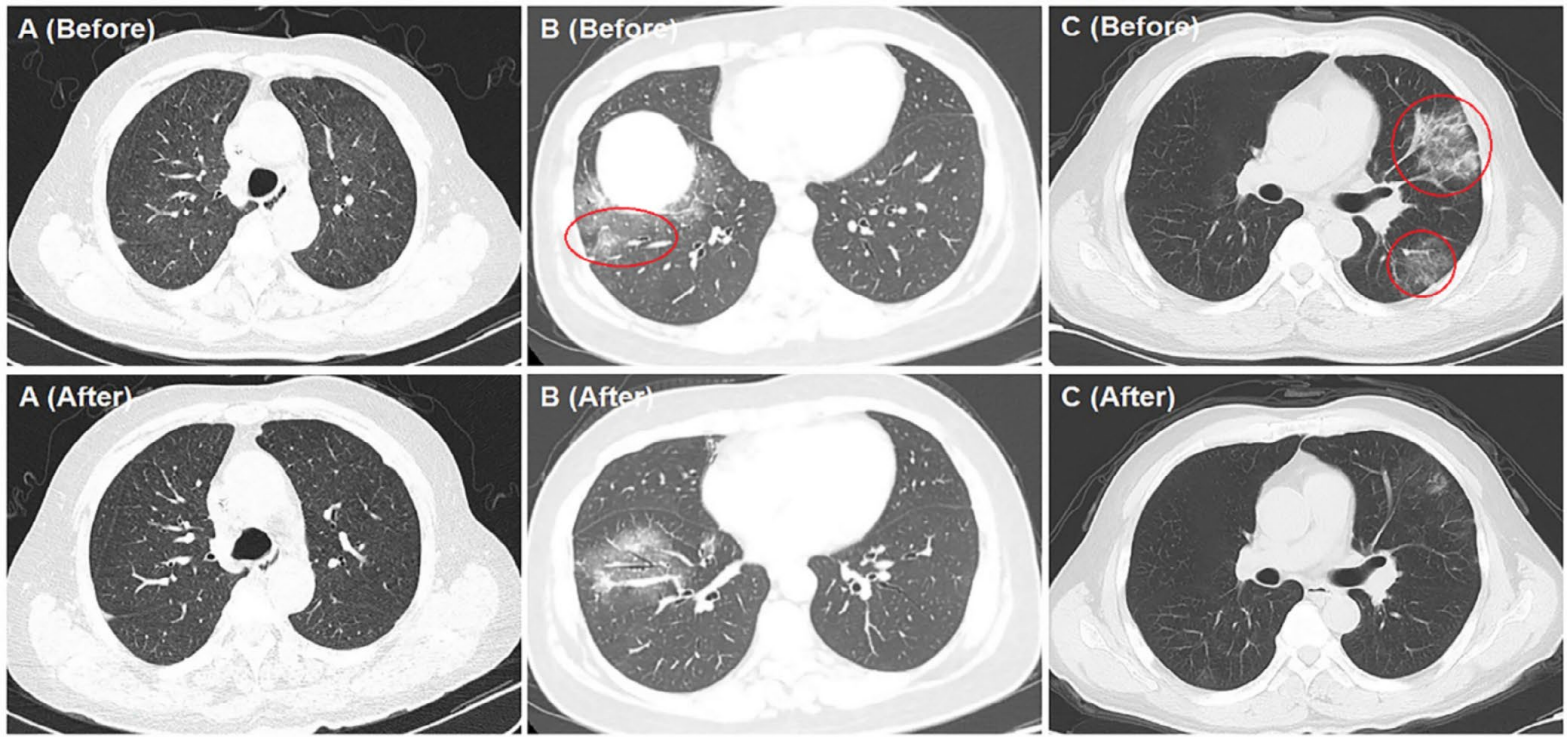
Interstitial pneumonia in the three patients before and after COVID‐19 infection. (A, Before) Diffuse interstitial inflammation of both lungs. (A, After) Diffuse interstitial inflammation in both lungs was alleviated. (B, Before). Localized inflammation of right lung. (B, After). Localized inflammation in right lung was alleviated. (C, Before). Localized inflammation of left lung. (C, After). Localized inflammation in left lung was alleviated.

A 56‐year‐old female patient was diagnosed with follicular lymphoma (FL) (Grade II, Stage III A, FLIPI score: low‐risk group) in March 2023. After four cycles of G‐CHOP (Obinutuzumab, Cyclophosphamide, Doxorubicin, Vincristine, Prednisone), she was evaluated for complete remission (CR) by Positron Emission Tomography‐Computed Tomography (PET‐CT). She developed a COVID‐19 infection diagnosed by a positive test for SARS‐CoV‐2 on pharyngeal swabs (RT‐PCR) in March 2023 and made a quick recovery. She had symptoms of persistent fever, coughing, and shortness of breath for 2 weeks in May 2023. The oxygen saturation was 85%–95% without oxygen inhalation. She tested negative for SARS‐CoV‐2 by pharyngeal swabs. Then she was ruled out for bacterial, viral, fungal, and other pathogen infections. Interleukin (IL)‐6 was 22.32 pg/mL (0–5 pg/mL), and C‐reactive protein (CRP) was 14.45 mg/L (0–20 mg/L) in peripheral blood. The expression of CD19 in B lymphocyte was 0.02%, and the globulin was 16.5 g/L. The percentages and absolute values of CD8^+^ T cells and CD4^+^ T cells are listed in Table 1. Computed tomography (CT) on admission indicated interstitial pneumonia in both of her both lungs (Figure 1A, Before). After 1 week, the symptoms did not resolve, and she was persistently negative for SARS‐CoV‐2 on pharyngeal swabs, so she underwent a bronchial examination. Her COVID‐19 infection was confirmed by metagenomics next‐generation sequencing (mNGS) of bronchoalveolar lavage fluid (BALF). The level of IL‐6 in BALF was 168.19 pg/mL, while that in peripheral blood on the same day was 29.31 pg/mL. She received molnupiravir and methylprednisolone while stopping other anti‐infection treatments; then her symptoms were relieved quickly in 2 weeks (Figure 1A, After). But the patient temporarily discontinued therapy for FL because of persistent interstitial inflammation. After 19 months without any treatment for FL, she was in a constant state of CR.

**TABLE 1:** The percentages and absolute values of CD8^+^ T cells and CD4^+^ T cells.

	Percentages (%)			Absolute values(/μL)		
	CD3^+^	CD3^+^CD4^+^	CD3^+^CD8^+^	CD3^+^	CD3^+^CD4^+^	CD3^+^CD8^+^
Case 1	89.57	29.95	65.09	1505.21	463.17	1008.03
Case 2	93.41	28.45	62.21	1468.34	451.27	986.18
Case 3	91.29	32.11	57.64	1736.19	595.74	1068.41

*Note:* The expression of cell antigens, cell size and the amount of intracellular granulation were detected by fluorescein labeled monoclonal antibodies and flow cytometry, thereby identifying normal and abnormal cells. The monoclonal antibodies and the corresponding reagents were from BD Company of the United States. The instrument used was a BECKMAN cytoflex flow cytometer, and the analysis was conducted using cytexpert software. The reference ranges for the percentage of CD3^+^ cells is 56% to 86%, the percentage of CD3^+^CD4^+^ cells is 33% to 58%, the percentage of CD3^+^CD8^+^ cells is 13% to 39%. The reference ranges for the absolute values(/μL) of CD3^+^ cells is 19,600–81,700, the absolute values(/μL) of CD3^+^CD4^+^ cells is 1155 to 5510, the absolute values(/μL) of CD3 + CD4+ cells is 455 to 3705.

### 2.2 Case 2

A 53‐year‐old female patient was diagnosed with FL (Grade II, Stage IV B, FLIPI score: high‐risk group) in November 2021. After four cycles of G‐CHOP, she was evaluated for partial remission (PR) by PET‐CT. Unfortunately, she was evaluated for PR after the following four cycles of G‐Gemox (Gemcitabine and Oxaliplatin) and 4 months of PI3Kδ inhibitor (Linperlisib) therapy. She was first developed a COVID‐19 infection diagnosed by positive for SARS‐CoV‐2 on pharyngeal swabs in January 2023 and had a quick recovery. However, she had symptoms of persistent fever, hypoxemia, and cough for 10 days in June 2023. The oxygen saturation of the patient was 88%–96% without oxygen inhalation. She tested negative for SARS‐CoV‐2 by pharyngeal swabs and had ruled out of infection by other pathogens. The IL‐6 was 21.67 pg/mL, the CRP was 24.64 mg/L, the expression of CD19 in B lymphocytes was 0.04%, and the serum immunoglobulin was 14.6 g/L. The percentages and absolute values of CD8^+^ T cells and CD4^+^ T cells are listed in Table 1. CT on admission indicated interstitial pneumonia and small nodules in both her both lungs (Figure 1B, Before). She was ruled out of infections by bacterial, influenza virus, fungal, and other pathogens. After failing to respond to anti‐infection treatment and testing persistently negative for SARS‐CoV‐2 on pharyngeal swabs, she underwent a bronchial examination. Her COVID‐19 infection was also confirmed by mNGS of BALF. The level of IL‐6 in BALF was 120.66 pg/mL, while that in peripheral blood on the same day was 25.01 pg/mL. She received molnupiravir and methylprednisolone, and her symptoms were relieved 1 month later (Figure 1B, After). The patient was temporarily discontinued the FL therapy due to recurrent infections, including COVID‐19 infection. However, what is surprising is that after the COVID‐19 infection, without any treatment for FL, she was evaluated with CR by PET‐CT after this COVID‐19 infection. So far, she has been in CR condition for 18 months without any treatment for FL.

### 2.3 Case 3

A 70‐year‐old male patient was diagnosed with FL (Grade II, Stage IV A, FLIPI score: high risk group) in 2022. He received CD19 CAR‐T cell therapy and obtained CR in December 2023. He developed a COVID‐19 infection diagnosed by positivity for SARS‐CoV‐2 on pharyngeal swabs and relieved quickly in January 2024. He was hospitalized again due to persistent fever, severe hypoxemia with cough, and had an oxygen saturation at 80%–85% without oxygen inhalation in March 2024. He had a negative result for SARS‐CoV‐2 by pharyngeal swabs. The interleukin IL‐6 was 35.30 pg/mL, the CRP was 32.02 mg/L, expression of CD19 in B lymphocytes in peripheral blood was 0.01%, and the serum immunoglobulin was 12.3 g/L. The percentages and absolute values of CD8^+^ T cells and CD4^+^ T cells are listed in Tables 1 and 2. CT on admission indicated interstitial pneumonia in both lungs (Figure 1C, Before). He underwent a bronchial examination because he was unresponsive to treatment and was ruled out for infections from bacterial, influenza virus, fungal, and other pathogens. His COVID‐19 infection was confirmed by mNGS of BALF as well. The level of IL‐6 in BALF was 265.12 pg/mL, while that in peripheral blood on the same day was 29.78 pg/mL. After the combination therapy of molnupiravir, methylprednisolone, and Ruxolitinib, his symptoms were relieved 1 month later. Meanwhile, the CT examination showed that his interstitial pneumonia was significantly absorbed 2 months later (Figure 1C, After). To date, he has remained in CR condition for 9 months without any treatment for FL.

**TABLE 2:** The counts and percentages of B cell, the levels of serum immunoglobulin and IgG and the sequence number of COVID‐19 in BALF.

	B‐cell counts (cells/μL)	Percentage of B‐cell (%)	Globulin levels (g/L)	IgG levels (g/L)	COVID‐19 in BALF (sequence number)
Case 1	0.08	0.02	16.5	12.3	15,537
Case 2	0.36	0.04	14.6	10.8	6602
Case 3	0.02	0.01	12.3	9.3	11,569

## 3 Discussion

Anti‐CD20 monoclonal antibody and CD19 CAR‐T cell therapy had been effective in patients with FL or relapsed/refractory (R/R) FL. Immunocompromised states caused by B lymphocyte deficiency and hypogammaglobulinemia after these therapies are high risks for severe COVID‐19 infection [6, 7]. The three patients with FL who received such therapy developed persistent fever, cough, and hypoxemia. Their infections were characterized by differences in viral load in the upper and lower respiratory tracts, with the upper respiratory tract showing negative SARS‐CoV‐2 by pharyngeal swabs and the lower respiratory tract showing positive confirmed by mNGS of BALF.

High‐dimensional flow cytometry analysis suggests that CD8^+^ T cells are the key prognostic determinants during acute SARS‐CoV‐2 infection [8]. Although CD8^+^ T cells are essential for acute infection, they are not sufficient to ensure timely clearance of the virus. CD4^+^ T cells or B cells play an important role in ultimately clearing the virus and preventing viral infection [9, 10]. COVID‐19 infection characterized by differences in viral load in the upper and lower respiratory tracts has been reported [11]. Although our three patients had B lymphocyte deficiency and hypogammaglobulinemia, their percentages and absolute values of CD8^+^ T cells and CD4^+^ T cells were normal at this time. These clinical features are not completely consistent with previous literature reports. Autopsy study showed that although the virus has been cleared in the nasopharynx, remaining SARS‐CoV‐2 virus was found in pneumocytes [12]. SARS‐CoV‐2 virus remains in the lung cells of immunocompromised patients, leading to the recurrence of COVID‐19 infection.

In addition, there were studies revealing that this COVID‐19 infection was driven by immunoreaction and no more by virus replication [13, 14]. Studies [15, 16] have shown that the number of Th2 cells in the blood of patients with SARS‐CoV‐2‐induced diseases decreases. Patients with severe COVID‐19 infection promote a hyperactive Th1 cellular state, rendering a systemic anti‐viral response due to a high state of immune activation. The hyperactive cells will turn into an exhausted state, which results in the reduction of their anti‐tumor activity.

Another relevant indicator, higher levels of IL‐6 in peripheral blood have been positively correlated with severe COVID‐19 infection and associated with poor outcomes in patients with COVID‐19 infection [17, 18, 19, 20]. The correlation between elevated levels of IL‐6 and mechanical ventilation is a possible pathogenesis of severe COVID‐19 infection [21]. These three FL patients with COVID‐19 infection we reported had a characteristic that the level of IL‐6 in peripheral blood was lower than that in BALF at the same time. Differential expression of IL‐6 was consistent with the differential expression of viral load in the upper and lower respiratory tracts. IL‐6 levels in BALF were significantly higher than that in peripheral blood, ranging from 13–37 times higher than in blood samples. The systemic levels of cytokines caused by COVID‐19 infection might be lower than in sepsis, but the local response is more intense [22]. Therefore, cytokine levels in circulation may not accurately reflect those in local tissue. This result might suggest that when the level of IL‐6 in peripheral blood is inconsistent with clinical symptoms, it is necessary to perform pathogen detection by mNGS of BALF immediately.

For the diagnosis of COVID‐19 infection, the collection of pharyngeal swabs is rapid, simple, and safe, and is feasible for routine laboratory diagnosis and monitoring of SARS‐CoV‐2. A study reported that mNGS of BALF maintains a high positive rate in the severe COVID‐19 infection, and the viral loads and positive rates in COVID‐19 infection patients gradually decreased during disease progression [23]. Studies found that viral shedding in the lower respiratory tract is far longer than the previous findings based on upper respiratory samples [23, 24]. Although viral RNA was negative in the upper respiratory tract, it was also positive in BALF during disease progression. The mNGS of BALF has been reported to improve the accuracy of diagnosis and monitoring of viral shedding in severe COVID‐19 infection patients. Therefore, for hematologic malignancy patients with respiratory symptoms who have negative SARS‐CoV‐2 by pharyngeal swabs, the use of mNGS of BALF could improve the diagnosis of COVID‐19 infection, provide timely treatment, and increase the survival rate.

Interestingly, another characteristic that we observed in these patients was that they maintained a durable remission of their FL without any therapy after this particular COVID‐19 infection. Some studies suggested that COVID‐19 infection triggers an immune response that induces a local flare phenomenon which was followed by the abscopal effect [25]. We need to further observe and verify in more similar patients.

In conclusion, in our case series, COVID‐19 infection was characterized by differences in viral load in the upper and lower respiratory tracts. In immunocompromised patients, especially FL patients with B lymphocyte deficiency, hypogammaglobulinemia, and low expression of IL‐6 in peripheral blood, inconsistent with clinical infection symptoms. The mNGS of BALF was performed as soon as possible to confirm the diagnosis of COVID‐19 infection. The small number of cases is the main limitation of our observation results, which needs further observation and verification.

## Author Contributions

Q.D.: concept and design. C.L. and Y.S.: drafted or revised the manuscript. S.N., T.Z., X.L., and R.C.: clinical work and acquisition of data. S.N.: analysis and interpretation of data. Q.D.: writing, review, and/or revision of manuscript. All authors had full access to the data, contributed to the study, approved the final version for publication, and take responsibility for its accuracy and integrity.

## Ethics Statement

The patients were managed in accordance with the Declaration of Helsinki and provided informed consent for publication.

## Conflicts of Interest

All authors have completed the ICMJE uniform disclosure form. The authors have no conflicts of interest to declare.

## Data Availability

The data that support the findings of this study are available on request from the corresponding authors. The data are not publicly available due to privacy or ethical restrictions.
